# Joint association of triglyceride glucose index (TyG) and body roundness index with the risk of periodontitis: a cross-sectional study

**DOI:** 10.3389/fnut.2025.1642112

**Published:** 2025-10-13

**Authors:** Liang Wang, Jia Liu, Junjie Wang, Pan Liu, Qin Zhao, Yu Wang, Hongyu Zhao, Xiao Zhu, Shan Liu, Jinqiang Zhang

**Affiliations:** ^1^Xiangya Nursing School, Central South University, Changsha, China; ^2^Health Management Medicine Center, The Third Xiangya Hospital, Central South University, Changsha, Hunan, China; ^3^Nursing Department, The Third Xiangya Hospital, Central South University, Changsha, Hunan, China; ^4^Department of Geo-Informatics, Central South University, Changsha, China; ^5^School of Automation, Central South University, Changsha, China; ^6^College of Nursing and Public Health, Adelphi University, Garden City, NY, United States; ^7^Department of Clinical Psychology, The Third Xiangya Hospital, Central South University, Changsha, Hunan, China

**Keywords:** body roundness index, triglyceride-glucose index, periodontitis, cross-sectional study, body fat

## Abstract

**Background:**

Periodontitis is a prevalent chronic inflammatory non-infectious condition, primarily induced by subgingival bacteria. The aim of this study was to investigate the interaction between body roundness index (BRI) and TyG index on periodontitis and to explore whether the connection was associated with sex.

**Study design:**

Cross-sectional study.

**Methods:**

This cross-sectional study included 261,454 participants between 2017 and 2024. The associations of the TyG index and BRI with periodontitis risk were investigated via logistic regression analysis and restricted cubic splines. Subgroup analysis was used to explore potential differences. A sensitivity analysis was conducted using multivariate regression to evaluate the associations between TyG-related indicators and periodontitis.

**Results:**

Among the 261,454 participants, 40,991 individuals were diagnosed with periodontitis. Individuals with high TyG and high BRI (TyG > 8.60 and BRI > 3.19) had the highest risk of developing periodontitis, suggesting a synergistic effect. Next, we multiplied these two metrics to establish TyG-BRI. TyG-BRI was nonlinearly positively correlated with the risk of periodontitis, with the highest quartile having a more significant effect on the risk of periodontitis compared to the lowest quartile (OR = 1.47; 95% CI: 1.40, 1.53). The TyG-BRI index had a significant effect on periodontitis in those less than 60 years of age, female, and non-smokers, and this effect was particularly prominent in women aged 30–50 years. Sensitivity analysis showed that the associations between the correlation indices (TyG index, BRI, and TyG-BRI) and periodontitis are statistically significant.

**Conclusion:**

Overall, the TyG-BRI index can be used as a predictor of periodontitis risk. The link was strongest in individuals under 60 years, women, and non-smokers, suggesting potential roles of age-related metabolic changes, estrogen, and smoking-induced systemic inflammation in modulating this relationship. In the future, this mechanism needs to be further verified in combination with the levels of sex hormones.

## Introduction

Periodontitis is a prevalent chronic inflammatory non-infectious condition, primarily induced by subgingival bacteria (1). It is marked by the progressive degradation of periodontal tissues, evidenced by the creation of periodontal pockets, loss of alveolar bone, and hemorrhaging gums (2). The prevalence of periodontitis in China varies from 1% to 69% and escalates progressively with age (3, 4). Furthermore, the prevalence of periodontitis in China is substantial and on the rise (5). This scenario underscores the critical public health issue of periodontitis and the pressing necessity for enhanced research and focus. The global frequency of periodontitis is notably high, with severe instances accounting for 11.2% (6). Periodontitis results in tooth loss, malnutrition, and several health complications (7). Consequently, it is crucial to acknowledge the elevated prevalence of periodontitis, particularly in China, to enhance public health.

Furthermore, periodontitis is significantly linked to a heightened risk of chronic conditions, including metabolic syndrome and diabetes, with insulin resistance (IR) potentially serving as a critical element in this relationship (8–10). Traditional methods of measuring IR are intricate; therefore, the researchers devised more straightforward assessment tools, such as the triglyceride glucose (TyG) index (11). The TyG index, which estimates IR by measuring triglyceride (TG) and fasting blood glucose levels, has been shown to be significantly associated with periodontitis risk (12). According to research, being overweight may make the dysregulation of periodontal microbiota worse by increasing the release of inflammatory mediators and affecting insulin resistance (13–15). To measure fat distribution more comprehensively, Thomas et al. proposed a new indicator, the body roundness index (BRI) (16). Unlike waist-to-height ratio (WHtR), the BRI ellipsoid model can more accurately reflect the geometric changes of visceral fat accumulation. Studies have shown that BRI is superior to other traditional anthropometric measures in predicting the risk of a variety of clinical endpoints, such as cardiometabolic disease, cancer, and death (17–19). Simultaneously, BRI is intricately associated with insulin resistance (20). However, at present, for the synergy between BRI and TyG indices, further verification and in-depth exploration are still needed.

Recent research has shown that there is an independent association between a single component and its combined components of gingival bleeding (BOP) in women and metabolic syndrome (21). This discovery strongly supports the necessity of further exploring the correlation between biological sex and periodontitis. Sex is also a key factor in obesity outcomes. Compared with men, women are more often obese but have fewer metabolic complications (22). Central obesity in women has the strongest correlation with CRP, while the fat percentage of systemic inflammation in men is a stronger predictor (23). The basic aspects of metabolic homeostasis are regulated differently in men and women, and may affect the risks of obesity and insulin resistance, both of which are risk factors for periodontitis (24). Whether the TyG-BRI index has different effects in different genders still needs further exploration. In view of the above situation, it is very necessary to conduct subgroup discussions on the association between the TyG-BRI index and periodontitis.

Therefore, this study aims to investigate the synergistic effect of TyG and BRI on periodontitis risk, develop a composite index to explore their integrated relevance, and identify subgroup-specific associations that may inform targeted screening strategies.

## Methods

### Study population and design

**Figure 1:**
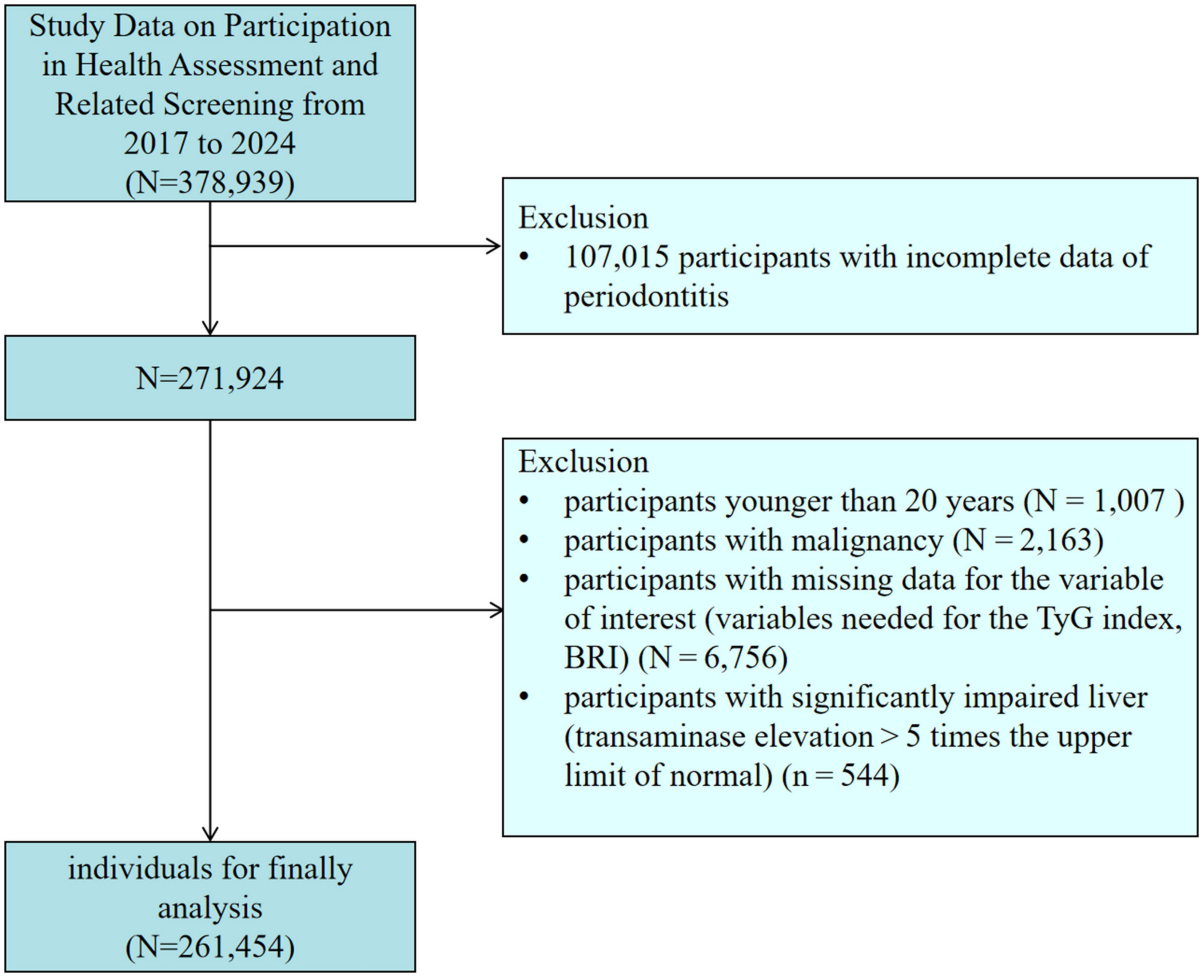
Flowchart of participants in this study. BRI, body roundness index; TyG, triglyceride-glucose.

This research utilized data from the database of the Third Xiangya Hospital at Central South University. The database is a cross-sectional survey administered by the Xiangya Third Hospital Center of Central South University. The Ethics Committee of the Third Xiangya Hospital approved the survey (No: 25118), and informed consent was acquired. The data collection for this study comprised two components: laboratory testing and the completion of a health assessment questionnaire via the website^1^. All blood sample collections strictly followed the Chinese “WS/T2252002” standard. After the subjects fasted for ≥8 h, venous blood was collected using vacuum blood collection tubes. Blood glucose was detected by the hexokinase method (in compliance with WS/T 352–2011), and triglyceride was detected by the glycerophosphate oxidase-peroxidase method (in compliance with WS/T 356–2011). The testing equipment is [Mindray BS-2000M fully automatic Biochemical Analyzer]. Each batch of testing includes quality control products provided by the National Health Commission Clinical Laboratory Center (batch Number: GBW09193). Quality control was strictly observed throughout the process. Data was collected by qualified and experienced medical staff in accordance with standards. The data was rechecked by two people and de-identified. Figure 1 illustrates the flow chart for the inclusion and exclusion of participants. We excluded (1) individuals who did not receive an oral examination (*n* = 107,015); (2) participants younger than 20 (*n* = 1,007); (3) participants with malignancy (*n* = 2,163); (4) participants with incomplete data on pertinent variables (TyG index, BRI) (*n* = 6,756); (5) participants with significantly impaired liver (transaminase elevation > 5 times the upper limit of normal) (*n* = 544). The conclusive sample of participants incorporated in this investigation comprised 261,454 individuals.

### Assessment of periodontitis

Full dental examination (excluding third molar) by a trained professional. Measurements were taken at 6 sites on each tooth. The case definition for a patient with periodontitis is defined as interdental clinical attachment loss detectable at ≥ 2 non-adjacent teeth, or buccal or oral clinical attachment loss ≥ 3 mm with pocketing ≥ 3 mm detectable at ≥ 2 teeth (25). After a series of assessments, individuals without periodontitis were assigned to the non-periodontitis group.

### The calculation of insulin resistance indices and obesity-related parameters

TyG index = Ln [fasting triglyceride (mg/dL) × fasting glucose (mg/dL)/2] (26);

The Body Roundness Index (BRI) was calculated using the formula: BRI = 364.2–365.5 × √(1 – [waist circumference in centimeters/2π]^2^/[0.5 × height in centimeters]^2^) (16). Waist circumference and height were measured at the examination centers. WHtR was determined by WC (cm)/height (cm) (27).

### Definitions of covariates

The covariates included age, sex, knowledge, body mass index (BMI) and some laboratory findings. Sex was specifically classified as male or female. Age was divided into two groups with a threshold of 60. The knowledge variable, which indicates whether participants were actively acquiring information about periodontitis, was categorized into two levels: “No” and “Yes.” BMI “<25” was considered within the normal weight range, while BMI “≥25” was defined as overweight/obese (28). Laboratory parameters included: systolic blood pressure (SBP), diastolic blood pressure (DBP), total serum protein (TP), serum albumin (ALB), serum globulin (GLB), blood uric acid (BUA), Total Bilirubin (TBIL), high-density lipoprotein-cholesterol (HDL-C), and low-density lipoprotein-cholesterol (LDL-C).

### Statistical analysis

The study used R software (4.3.1) for data processing and statistical analysis. Categorical variables were described using frequencies (*n*) and percentages (%), and chi-square tests were used for between-group comparisons. Used column-wise median imputation to preserve original distribution. Since none of the continuous variables in this study conformed to normal distribution, they were expressed using medians and interquartile range (IQRs), and intergroup comparisons were made using the Mann–Whitney *U* test.

Logistic regression analysis was employed to investigate the associations of the TyG index and BRI with periodontitis. The covariate selection strategy aimed to control confounding effects by integrating theoretical frameworks with univariate statistical screening. Specifically, all measured covariates first underwent univariate logistic regression, and variables with *p* < 0.05 were shortlisted into a candidate pool. Final covariates for Model 2 were determined by synthesizing literature-supported biological plausibility and collinearity analysis results. Model 1 adjusted for age, sex, diabetes (29), and high-density lipoprotein-cholesterol (30). Model 2 adjusted for Model 1 plus Knowledge (31), BMI ≥ 25 (32), systolic blood pressure (33), diastolic blood pressure (33), blood uric acid (34), low-density lipoprotein-cholesterol (30), smoking (35), alcohol consumption (36). To assess multicollinearity in the multivariate logistic regression model, we calculated both tolerance and variance inflation factors (VIF). Variables with VIF > 5 (tolerance < 0.1) were excluded to ensure no significant collinearity among predictors. A restricted cubic spline model was used to determine the potential nonlinear associations of the TyG index and BRI with periodontitis. We explored whether there was a synergistic effect of the TyG index and BRI on periodontitis. Significance was set at *p* < 0.05 (*), *p* < 0.01 (**), and *p* < 0.001 (***). The classification accuracy of TyG-related indices was assessed through the implementation of a receiver operating characteristic (ROC) framework. The discriminative ability of these indices was quantified by calculating the area under the curve (AUC) metrics, with 1,000 bootstrap-resampled iterations employed to enhance the robustness of the estimates. Multiple subgroup analyses were performed to assess the robustness of the results of the correlation of TyG-BRI with periodontitis. To evaluate the robustness of the research results, a sensitivity analysis was conducted using the Chinese version of the BMI cut-off value (BMI ≥ 24) (37).

## Results

### The characteristics of study participants

Table 1 shows the baseline characteristics of the 261,454 study individuals according to TyG index quartiles. Among all participants, 40,991 individuals were diagnosed with periodontitis (15.68%). 10.3% of the participants were older than 60 years of age, and 54.0% were male in the gender distribution. Individuals in the higher quartile of the TyG index were older, tended to be male, and were more likely to be smokers. They were more likely to have diabetes than those in the lower TyG index quartile. Supplementary Table 1 summarizes the baseline characteristics of study individuals according to BRI quartiles.

**Table 1:** Characterization of participants according to the TyG index quartiles^a^.

Variable	Total, *n* = 261,454	TyG				*p*-value
		Q1 < 8.17, *N* = 65,778	Q2 8.17–8.59, *N* = 65,201	Q3 8.59–9.07, *N* = 65,537	Q4 > 9.07, *N* = 64,938	
Age (years), median (Q1–Q3)	41.00 [33.00;52.00]	35.00 [29.00;44.00]	41.00 [33.00;52.00]	45.00 [35.00;54.00]	46.00 [37.00;54.00]	<0.001
Age ≥ 60 years (*n*, %)	26,834 (10.26%)	3,307 (5.03%)	7,001 (10.74%)	8,860 (13.52%)	7,666 (11.81%)	<0.001
Sex (*n*, %)						<0.001
Male	141,094 (53.97%)	17,207 (26.16%)	30,525 (46.82%)	41,543 (63.39%)	51,819 (79.80%)	
Female	120,360 (46.03%)	48,571 (73.84%)	34,676 (53.18%)	23,994 (36.61%)	13,119 (20.20%)	
Knowledge (*n*, %)						<0.001
No	104,119 (39.82%)	25,316 (38.49%)	25,135 (38.55%)	26,228 (40.02%)	27,440 (42.26%)	
Yes	157,335 (60.18%)	40,462 (61.51%)	40,066 (61.45%)	39,309 (59.98%)	37,498 (57.74%)	
Diabetes (*n*, %)	6,871 (2.63%)	311 (0.47%)	715 (1.10%)	1,576 (2.40%)	4,269 (6.57%)	<0.001
Smoking (*n*, %)	62,735 (23.99%)	6,219 (9.45%)	11,825 (18.14%)	17,756 (27.09%)	26,935 (41.48%)	<0.001
Alcohol consumption (*n*, %)	76,250 (29.16%)	10,068 (15.31%)	15,425 (23.66%)	21,088 (32.18%)	29,669 (45.69%)	<0.001
BRI, median (Q1–Q3)	3.21 [2.50;3.96]	2.42 [1.96;2.98]	2.96 [2.40;3.62]	3.47 [2.88;4.12]	3.93 [3.36;4.57]	<0.001
Hips (cm), median (Q1–Q3)	94.00 [90.00;98.00]	91.0 [87.00;94.00]	93.0 [89.00;96.00]	95.0 [91.00;98.00]	96.0 [93.00;100.00]	<0.001
SBP (mmHg), median (Q1–Q3)	120 [110;131]	112 [104;122]	118 [108;128]	123 [113;133]	127 [118;136]	<0.001
DBP (mmHg), median (Q1–Q3)	73.0 [66.0;81.0]	68.0 [62.0;74.0]	71.0 [65.0;79.0]	75.0 [68.0;82.0]	79.0 [72.0;86.0]	<0.001
Pulse (bpm), median (Q1–Q3)	80.0 [73.0;88.0]	80.0 [72.0;88.0]	80.0 [72.0;88.0]	79.0 [72.0;87.0]	81.0 [73.0;89.0]	<0.001
TBIL (μmol/L), median (Q1–Q3)	12.4 [9.80;15.70]	12.5 [9.80;16.0]	12.5 [9.90;15.90]	12.4 [9.80;15.80]	12.1 [9.50;15.30]	<0.001
TP (g/L), median (Q1–Q3)	72.7 [70.1;75.3]	71.8 [69.4;74.4]	72.3 [69.8;74.8]	72.8 [70.4;75.3]	73.8 [71.2;76.4]	<0.001
ALB (g/L), median (Q1–Q3)	46.6 [44.7;48.5]	46.2 [44.4;48.1]	46.3 [44.5;48.2]	46.6 [44.8;48.5]	47.2 [45.3;49.0]	<0.001
GLB (g/L), median (Q1–Q3)	26.0 [23.8;28.3]	25.5 [23.4;27.7]	25.8 [23.7;28.1]	26.1 [23.9;28.4]	26.5 [24.3;29.0]	<0.001
Creatinine (μmol/L), median (Q1–Q3)	70.0 [58.0;82.0]	61.0 [54.0;72.0]	68.0 [57.0;81.0]	74.0 [61.0;85.0]	77.0 [67.0;87.0]	<0.001
BUA (μmol/L), median (Q1–Q3)	328 [270;392]	275 [237;325]	310 [261;367]	346 [292;402]	384 [330;441]	<0.001
Fasting triglyceride (mmol/L), median (Q1–Q3)	5.25 [4.92;5.65]	4.98 [4.71;5.26]	5.18 [4.90;5.49]	5.34 [5.02;5.71]	5.63 [5.22;6.28]	<0.001
fasting glucose (mmol/L), median (Q1–Q3)	1.27 [0.86;1.97]	0.69 [0.58;0.78]	1.06 [0.95;1.18]	1.57 [1.40;1.78]	2.76 [2.28;3.74]	<0.001
HDL_c (mmol/L), median (Q1–Q3)	1.31 [1.13;1.52]	1.51 [1.33;1.71]	1.38 [1.21;1.57]	1.25 [1.11;1.43]	1.12 [0.99;1.27]	<0.001
LDL_c (mmol/L), median (Q1–Q3)	2.82 [2.32;3.36]	2.57 [2.17;3.02]	2.89 [2.42;3.38]	3.06 [2.56;3.59]	2.79 [2.17;3.41]	<0.001
BMI, kg/m^2^, median (Q1–Q3)	23.70 [21.50;26.00]	21.40 [19.90;23.30]	23.00 [21.10;24.90]	24.40 [22.60;26.40]	25.80 [24.00;27.80]	<0.001
BMI ≥ 25 (*n*, %)	91,070 (34.83%)	6,976 (10.61%)	16,094 (24.68%)	27,655 (42.20%)	40,345 (62.13%)	<0.001
WHtR	0.49 [0.45;0.53]	0.45 [0.42;0.48]	0.48 [0.45;0.51]	0.51 [0.47;0.54]	0.53 [0.50;0.56]	<0.001

BRI, body roundness index; TyG, triglyceride-glucose; SBP, systolic blood pressure; DBP, diastolic blood pressure; TP, total serum protein; ALB, serum albumin; GLB, serum globulin; BUA, blood uric acid; TBIL, Total Bilirubin; HDL-C, high-density lipoprotein-cholesterol; LDL-C, low-density lipoprotein-cholesterol; WHtR, waist-to-height ratio; BMI, body mass index.

^a^The survey data was expressed as the median (IQRs) of the continuous variables and the unweighted frequency (weighted proportion) of the categorical variables.

### Association between the TyG index and periodontitis

**Figure 2:**
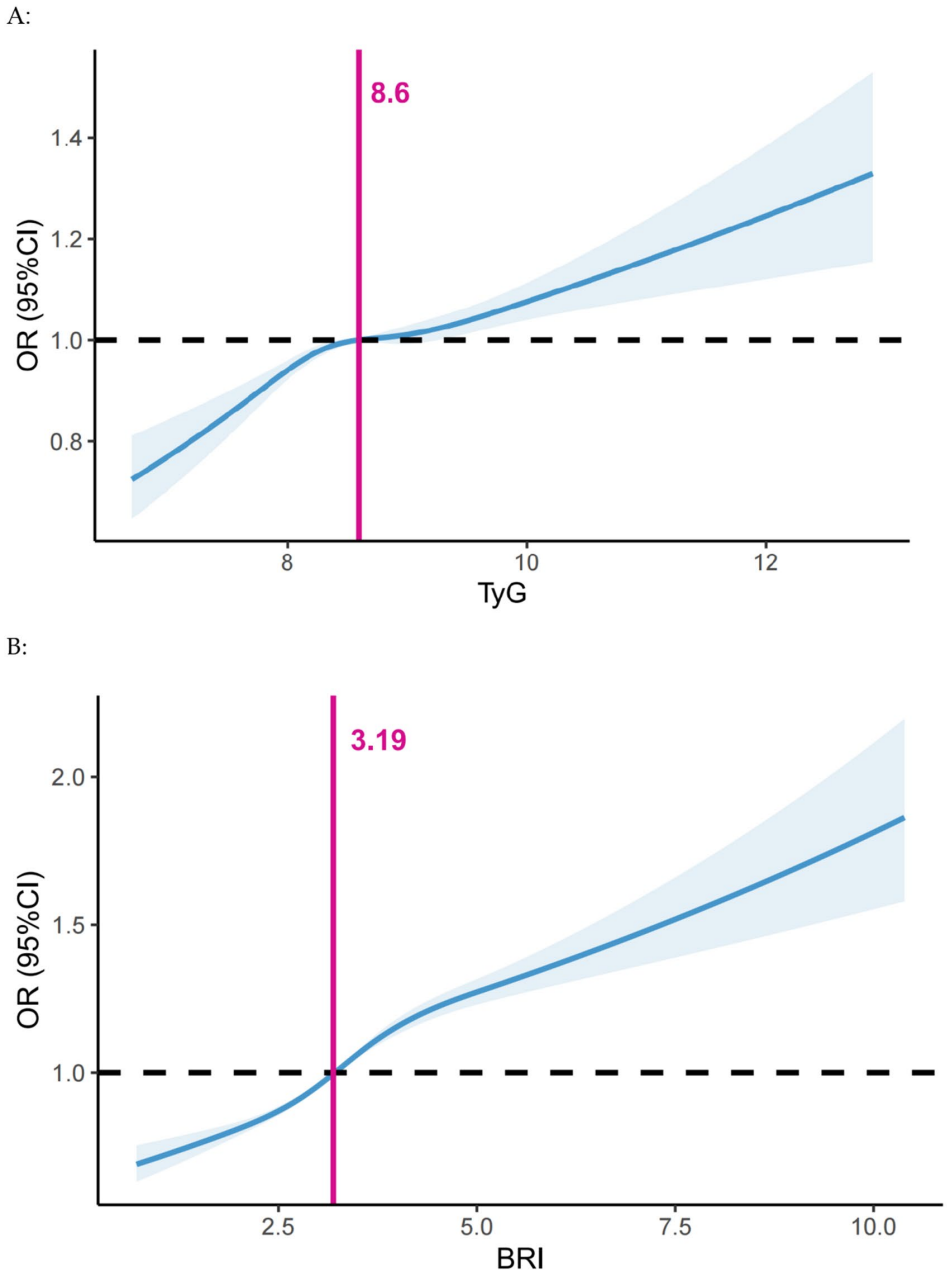
Dose–response associations of **(A)** the TyG index and **(B)** BRI with periodontitis assessed by restricted cubic spline model. Adjusted for Model 2 in the logistic analysis. BRI, body roundness index; CI, confidence interval; OR, Odds Ratio; TyG, triglyceride-glucose.

The results of the univariate logistic regression analyses are shown in Supplementary Figure 1. The risk of developing periodontitis increased with age, and men, smokers, and diabetics were more likely to develop periodontitis. A positive understanding of periodontitis was a relevant protective factor. Before conducting logistic regression, we performed a multicollinearity analysis on the independent variables in the model, showing that VIF < 5, suggesting that there was no significant multicollinearity in our model (Supplementary Table 2). As shown in Supplementary Table 3, independent of covariate adjustment, the risk of periodontitis increased with increasing TyG index (*p* < 0.001). In the fully adjusted model (model 2), TyG index (OR = 1.08; 95% CI: 1.06, 1.10; *p* < 0.001) was considered a risk factor for periodontitis. Restricted cubic spline analysis showed a significant positive linear relationship between TyG index and periodontitis. The risk of developing periodontitis was significantly higher when the TyG index exceeded 8.60 (Figure 2A).

### Association between the BRI and periodontitis

Supplementary Table 4 illustrates that the risk of periodontitis escalated with a rising BRI (*p* < 0.001), independent of covariate adjustments. In the fully adjusted model (model 2), BRI (OR = 1.15; 95% CI: 1.13, 1.16; *p* < 0.001) was a risk factor for periodontitis. Restricted cubic spline analysis revealed a significant positive linear relationship between BRI and periodontitis. The risk of periodontitis was elevated when the BRI index surpassed 3.19 (Figure 2B).

### The synergistic effect of the TyG index and BRI on periodontitis

The joint effect of BRI and TyG index on the risk of periodontitis was assessed. The results showed that the interaction term between BRI and TyG index was statistically significant (*p* < 0.001). Individuals with high TyG and high BRI values (TyG > 8.60 and BRI > 3.19) had a higher risk of developing periodontitis than individuals with isolated high BRI values (BRI alone > 3.19) or isolated high TyG values (TyG > 8.60 only) and low TyG values (TyG < 8.60 and BRI < 3.19) (Supplementary Figure 2 and Supplementary Table 5). These results suggest that TyG index and BRI have a synergistic effect on periodontitis.

### Construction and analysis of TyG-BRI

**Figure 3:**
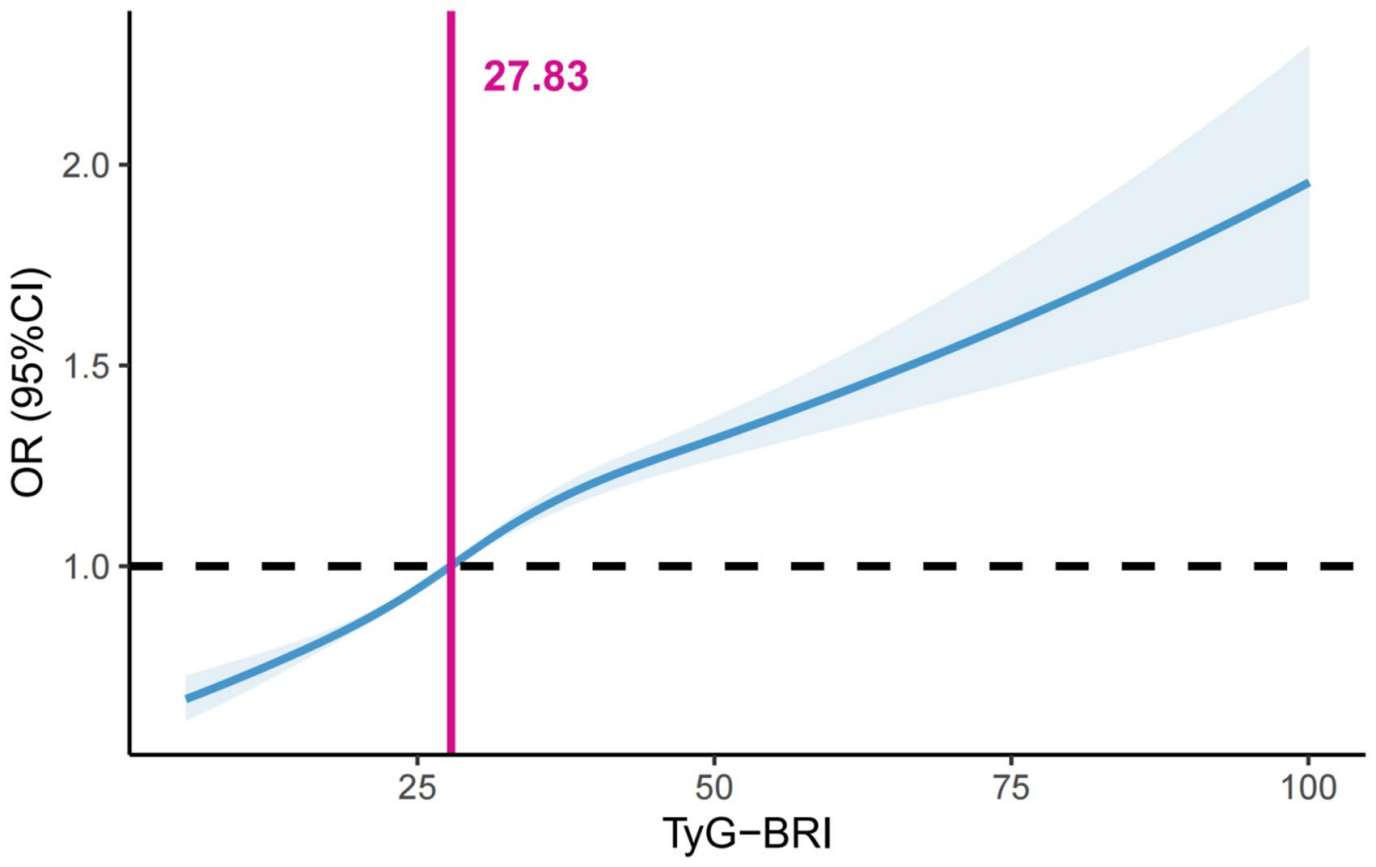
Dose–response associations of the TyG-BRI index with periodontitis assessed by restricted cubic spline model. BRI, body roundness index; TyG, triglyceride-glucose.

Considering the synergistic effect of the TyG index and the BRI on the risk of periodontitis, we multiplied these two indices to create the TyG-BRI. When analyzing the TyG-BRI as a continuous variable, the dose–response analysis showed a significant increase in the risk of periodontitis when the TyG-BRI exceeded 27.83 (Figure 3). In the fully adjusted model 2, TyG-BRI was categorised according to a threshold of 27.83 and was identified as a risk factor for periodontitis (OR = 1.21; 95% CI: 1.18, 1.24; *P* < 0.001) (Supplementary Table 6).

The receiver operating characteristic analysis for evaluating the TyG index and its obesity complex derivatives on the risk of periodontitis demonstrated a significant discriminatory ability. The predictive validity of TyG-BRI was evaluated, yielding an AUC of 0.5541 (95% CI: 0.5511–0.5571), demonstrating slightly higher discriminative ability than TyG-WHtR (AUC = 0.5533, 95% CI: 0.5503–0.5562), TyG (AUC = 0.5396, 95% CI: 0.5366–0.5426), BRI (AUC = 0.5529, 95% CI: 0.5499–0.5559), and WHtR (AUC = 0.5527, 95% CI: 0.5497–0.5557). DeLong’s test further validated the statistical significance of AUC differences. Compared with TyG, BRI, and WHtR, TyG-BRI showed significantly higher discriminative ability (all *p* < 0.001). The AUC of TyG-BRI was marginally higher than that of TyG-WHtR (*p* = 0.017), indicating a modest yet statistically significant improvement in predictive validity.

**Figure 4:**
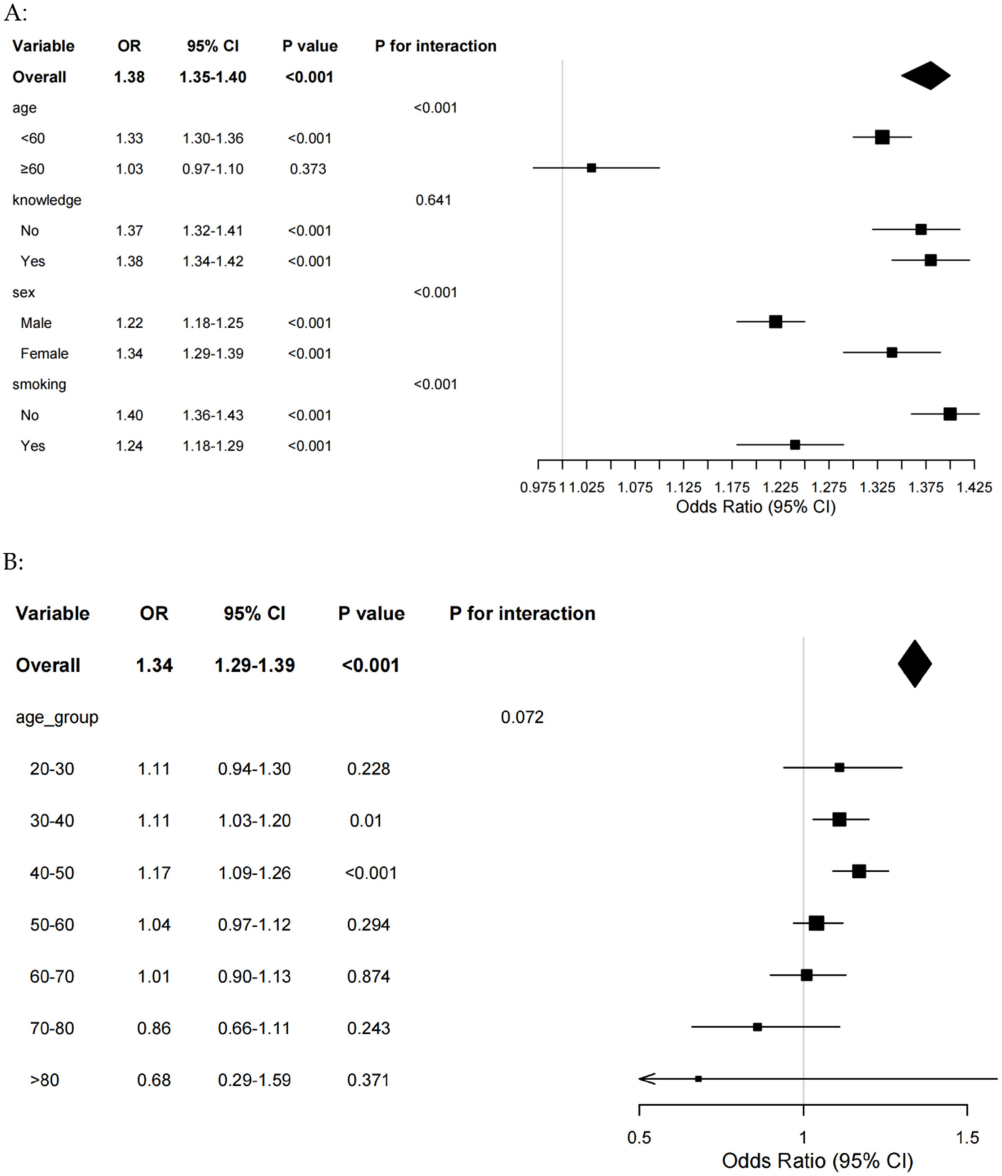
**(A)** Forest plot of subgroup analyses of TyG-BRI for periodontitis risk. **(B)** Forest plot of age subgroup analyses of TyG-BRI for periodontitis risk in female. BRI, body roundness index; TyG, triglyceride-glucose.

Further subgroup analyses were performed to explore whether TyG-BRI had a different effect in different genders. The results demonstrated that the association between TyG-BRI and periodontitis was significantly stronger in participants under 60 years of age, females, and non-smokers (Figure 4A). The interaction terms for age and TyG-BRI (*p* < 0.001), gender and TyG-BRI (*p* < 0.001), and smoking status and TyG-BRI (*p* < 0.001) were all statistically significant. To gain more insight into the role of the TyG-BRI index in the female population, an age-stratified analysis was performed on females (Figure 4B). The results of the analysis showed that the effect of the TyG-BRI index on periodontitis was significant in women aged 30–50 years.

To evaluate the robustness of the research results, a sensitivity analysis was conducted using the Chinese version of the BMI cut-off value (BMI ≥ 24). In all multivariate adjusted models analyzed continuously and categorically (based on quartiles), the associations between the correlation indices (TyG index, TyG-BRI, BRI) and periodontitis remained statistically significant (Supplementary Table 7).

## Discussion

This extensive cross-sectional study of the Chinese population explores the correlation between the BRI, TyG index, and the risk of periodontitis. The primary findings were as follows: (1) BRI, an essential indicator of central obesity, significantly increased the risk of periodontitis and functioned as an independent risk factor for the disease; (2) A synergistic interaction between BRI and the TyG index was observed among participants, collectively influencing the risk of periodontitis; (3) The TyG-BRI index had a significant effect on periodontitis in those less than 60 years of age, female, and non-smokers, and this effect was particularly prominent in women aged 30–50 years. Therefore, it is essential to acknowledge the influence of TyG-BRI index on the risk of periodontitis. Especially for women between the ages of 30 and 50, it is important to pay close attention to the TyG-BRI index. Meanwhile, through the TyG-BRI index, some interesting phenomena were found in this study.

The BRI index is a new validated index for detecting central obesity, which is important for the prevention of periodontitis and other chronic diseases (17, 38). Central obesity may lead to osteoporosis and interfere with osteoblast differentiation, which in turn increases the risk of periodontitis (39, 40). However, the BRI as a continuous indicator does not have clear cut-off values. This study employed restricted cubic spline analysis, which revealed a cutoff value of 3.19 for the nonlinear relationship between BRI and periodontitis, aligning with the cutoff value of BRI in other disease risks (41). Fat loss may have a significant therapeutic effect on the aforementioned diseases, such as dietary interventions, exercise interventions, and health education (42, 43). And acquisition of relevant knowledge was also a protective factor for reducing the risk of periodontitis. This implies that improving individuals’ knowledge and health literacy is equally crucial for effective obesity control and prevention of periodontitis.

Moreover, this study found that BRI and TyG exhibit synergistic effects in the development and progression of periodontitis. These two metrics reflect the metabolic status of the body from different perspectives, which together influence the risk of periodontitis (44, 45). Previous studies have also shown that the higher the TyG index, the greater the risk of periodontitis (46, 47). Restricted cubic spline analysis identified a TyG index threshold of 8.60, indicating that values ≥8.6 may reflect metabolic dysfunction potentially aggravating periodontal inflammation. Chronic inflammation from central obesity reduces insulin sensitivity and disrupts the oral immune microenvironment, thereby worsening periodontitis severity (13, 48). However, it has been shown that there may be a complex interaction between visceral adiposity and TyG, and that this interaction may manifest differently in different diseases (49). This phenomenon may be intricately linked to the metabolic pathways by which BRI and TyG index influence the risk of periodontitis (50). While single clinical indices offer advantages in practicality and interpretability, our study highlights the value of TyG-BRI, as evidenced by TyG-BRI’s statistically significant (though marginally higher) AUC compared to single indices. While the incremental improvements in AUC may appear modest, such exploratory research is crucial for advancing our understanding of optimal risk stratification strategies. When evaluating the clinical utility of a novel biomarker or risk factor, its potential should not be summarily dismissed solely on account of a suboptimal AUC value. This tension between simplicity and comprehensiveness embodies the core challenge of clinical biomarker research: balancing translational feasibility with mechanistic insight. Our findings underscore that such explorations, while requiring rigorous validation, are essential for advancing risk assessment frameworks beyond single-dimensional metrics. Therefore, the joint indicator BRI-TyG not only reveals the interaction between BRI and TyG, but also helps us to more accurately stratify the at-risk population and identify those individuals who are at high risk.

Men have a higher risk of developing periodontitis than women, a phenomenon that has been confirmed in several studies (51). Studies have shown that biological sex differences may influence the pathogenesis of periodontitis through a number of aspects, including gene expression on sex and autosomal chromosomes, the endocrine system, and the immune system (52–57). Nonetheless, there are some paradoxes, such as shown in this study, the effect of TyG-BRI on periodontitis was more pronounced in the female population. These paradoxical phenomena are partly due to differences in the distribution of adipose tissue between the sexes (22). In women, central obesity correlated most strongly with CRP, whereas in men, percent body composition fat was a stronger predictor of systemic inflammation (23). Androgens and estrogens are thought to play a key role in these gender differences and are thought to play a role in lipolysis, muscle metabolism, and satiety (58, 59). Meanwhile, a further subgroup analysis was conducted on the female population, and it was found that the influence of TyG-BRI on periodontitis was significant among women aged 30–50. This may be related to changes in hormone levels in women after the age of 30. Research based on multimodal measurements has identified a set of known and unknown age-related markers, determining that the ages of 30 and 50 are transitional periods of aging for women, during which there are significant changes in their hormone levels (60). The stratification results vary by age group, suggesting that in clinical practice, for the risk assessment and intervention of periodontitis, a one-size-fits-all approach should not be adopted. For females aged 30–50, more attention can be paid to TyG-BRI, and risk screening and early intervention can be carried out.

Although smokers have a higher risk of periodontitis, the effect of TyG-BRI on periodontitis is more pronounced in non-smokers. Smoking weakens the immune defenses of periodontal tissues, making it more difficult for the body to fight off periodontal infections (61). Nicotine may attenuate host immunity in early periodontitis, thereby increasing the risk of periodontitis (62, 63). In addition, there is a significant association between smoking and systemic inflammation (expressed as C-reactive protein levels) (24). Smoking causes vascular endothelial cells to constrict, reducing blood flow to the periodontal tissues, which decreases the number of immune cells and gingival sulcus fluid (64). This mechanism not only affects periodontal health, but may also have an impact on insulin resistance. However, smoking also leads to changes in gonadal hormone levels, particularly reduced androgen levels (65, 66). Considering that androgen levels are positively correlated with indicators of insulin resistance (67), future studies will allow testing this hypothesis and further elucidating the relationship between the relevance of dose-dependent effects of smoking.

Although individuals in the higher quartiles of BRI and TyG indices were older, the effect of TyG-BRI on periodontitis was more pronounced in those younger than 60 years. Aging can cause dysregulation of the inflammatory response, leading to increased expression of pro-inflammatory cytokines, which not only exacerbate local inflammation, but also affect the systemic metabolic state through blood circulation, leading to insulin resistance (68, 69). In younger individuals, metabolic dysregulation likely exerts a more direct impact on periodontal inflammation, whereas in older adults, chronic inflammatory states and multifactorial comorbidities may obscure this relationship (70). Another study also found that the association between TyG index and periodontitis was more pronounced in younger and middle-aged populations (12). Clinically, these results imply that age-stratified risk assessment using TyG-BRI could optimize early intervention strategies, particularly for younger adults.

This study offers significant insights but is not without limitations. Firstly, the observational design restricts a thorough examination of the causal relationships among the TyG index, BRI, and periodontitis, necessitating verification of the clinical practicability and predictive value of TyG-BRI through larger-scale prospective studies. Secondly, despite efforts to adjust for known confounders, eliminating the influence of other potential confounding variables remains challenging. Lastly, the study predominantly involved Chinese participants, which may limit the generalisability of the findings across different ethnic groups.

## Conclusion

In conclusion, based on the analysis of data from Chinese adult participants, the BRI and TyG indices not only correlate with the prevalence of periodontitis, but also interact with each other, potentially exacerbating the severity of periodontitis. Notably, our subgroup analysis suggests that TyG-BRI may possess enhanced predictive utility in populations (Female, younger adults, non-smokers), though this finding warrants validation in multiethnic, multicenter cohorts to address generalizability. Age-related metabolic changes, estrogen levels and systemic inflammation caused by smoking may play a potential role in regulating this relationship. While the clinical utility of the TyG-BRI index warrants further exploration and validation, this study provides valuable insights into the risk stratification strategy and lays the foundation for future research.

## Data Availability

The raw data supporting the conclusions of this article will be made available by the corresponding author upon reasonable request.

## Footnotes

^1^ https://new.selfhealth.com.cn/#/login
