# Supplementary material for: Joint association of triglyceride glucose index (TyG) and body roundness index with the risk of periodontitis: a cross-sectional study
Source: Front Nutr. 2025 Oct 13;12:1642112. doi: 10.3389/fnut.2025.1642112 (PMC12554622; doi:10.3389/fnut.2025.1642112)

**Supplementary Materials**

**Supplementary Table 1.** Characterization of participants according to the BRI quartiles^a^.

| **Variable** | **Total,n=261,454** | **BRI** | | | | **P value** |
| --- | --- | --- | --- | --- | --- | --- |
|  |  | **Q1 <2.50, N=65,863** | **Q2 2.50-3.21, N=65,433** | **Q3 3.21-3.96, N=65,094** | **Q4 >3.96, N=65,064** |  |
| Age(years) | 41.0 [33.0;52.0] | 33.0 [28.0;41.0] | 40.0 [33.0;50.0] | 45.0 [36.0;54.0] | 49.0 [39.0;57.0] | <0.001 |
| Age  ≥  60 years (n,%) | 26834 (10.3%) | 2006 (3.05%) | 4798 (7.33%) | 7978 (12.3%) | 12052 (18.5%) | <0.001 |
| Sex(n,%) |  |  |  |  |  | <0.001 |
| Male | 141094 (54.0%) | 17289 (26.2%) | 31298 (47.8%) | 43834 (67.3%) | 48673 (74.8%) |  |
| Female | 120360 (46.0%) | 48574 (73.8%) | 34135 (52.2%) | 221260 (32.7%) | 16391 (25.2%) |  |
| Knowledge(n,%) |  |  |  |  |  | <0.001 |
| No | 104119 (39.8%) | 26423 (40.1%) | 24933 (38.1%) | 25758 (39.6%) | 27005 (41.5%) |  |
| Yes | 157335 (60.2%) | 39440 (59.9%) | 40500 (61.9%) | 39336 (60.4%) | 38059 (58.5%) |  |
| Diabetes (n,%) |  |  |  |  |  | <0.001 |
| No | 254583 (97.4%) | 65561 (99.5%) | 64368 (98.4%) | 62956 (96.7%) | 61698 (94.8%) |  |
| Yes | 6871 (2.6%) | 302 (0.5%) | 1065 (1.6%) | 2138 (3.3%) | 3366 (5.2%) |  |
| Smoking (n,%) |  |  |  |  |  | <0.001 |
| No | 198719 (76.0%) | 57761  (87.7%) | 52590 (80.4%) | 46491 (71.4%) | 41877 (64.4%) |  |
| Yes | 62735 (24.0%) | 8102 (12.3%) | 12843 (19.6%) | 18603 (28.6%) | 23187 (35.6%) |  |
| Alcohol consumption (n,%) |  |  |  |  |  | <0.001 |
| No | 185204 (70.8%) | 55650 (84.5%) | 49355 (75.4%) | 42169 (64.8%) | 38030 (58.5%) |  |
| Yes | 76250 (29.2%) | 10213 (15.5%) | 16078 (24.6%) | 22925 (35.2%) | 27034 (41.5%) |  |
| TyG | 8.59 [8.17;9.07] | 8.13 [7.85;8.43] | 8.48 [8.14;8.86] | 8.79 [8.42;9.21] | 9.05 [8.66;9.49] | <0.001 |
| Hips (cm) | 94.0 [90.0;98.0] | 89.0 [86.0;92.0] | 92.0 [90.0;95.0] | 95.0 [92.0;98.0] | 99.0 [95.0;102] | <0.001 |
| SBP (mmHg) | 120 [110;131] | 112 [104;121] | 118 [108;128] | 123 [114;133] | 128 [119;138] | <0.001 |
| DBP (mmHg) | 73.0 [66.0;81.0] | 68.0 [62.0;74.0] | 71.0 [65.0;78.0] | 75.0 [68.0;83.0] | 79.0 [72.0;86.0] | <0.001 |
| Pulse (bpm) | 80.0 [73.0;88.0] | 82.0 [74.0;90.0] | 79.0 [72.0;87.0] | 79.0 [72.0;87.0] | 79.0 [72.0;87.0] | <0.001 |
| TBIL (μmol/L) | 12.4 [9.80;15.7] | 12.5 [9.80;16.0] | 12.3 [9.70;15.6] | 12.4 [9.80;15.8] | 12.2 [9.70;15.5] | <0.001 |
| TP (g/L) | 72.7 [70.1;75.3] | 72.5 [70.0;75.1] | 72.5 [70.0;75.1] | 72.8 [70.2;75.4] | 72.9 [70.3;75.6] | <0.001 |
| ALB (g/L) | 46.6 [44.7;48.5] | 46.7 [44.9;48.6] | 46.5 [44.7;48.4] | 46.6 [44.8;48.5] | 46.4 [44.5;48.3] | <0.001 |
| GLB (g/L) | 26.0 [23.8;28.3] | 25.7 [23.6;28.0] | 25.8 [23.7;28.1] | 26.0 [23.8;28.4] | 26.4 [24.1;28.8] | <0.001 |
| Creatinine (μmol/L) | 70.0 [58.0;82.0] | 61.0 [55.0;72.0] | 68.0 [57.0;81.0] | 75.0 [62.0;85.0] | 76.0 [65.0;86.0] | <0.001 |
| BUA (μmol/L) | 328 [270;392] | 276 [238;325] | 313 [262;372] | 351 [295;408] | 376 [321;434] | <0.001 |
| Fasting triglyceride (mmol/L) | 5.25 [4.92;5.65] | 5.03 [4.76;5.32] | 5.19 [4.89;5.52] | 5.33 [5.00;5.74] | 5.54 [5.14;6.09] | <0.001 |
| fasting glucose (mmol/L) | 1.27 [0.86;1.97] | 0.84 [0.65;1.13] | 1.15 [0.84;1.66] | 1.53 [1.07;2.26] | 1.88 [1.31;2.80] | <0.001 |
| HDL_c (mmol/L) | 1.31 [1.13;1.52] | 1.50 [1.31;1.70] | 1.35 [1.18;1.55] | 1.24 [1.09;1.42] | 1.17 [1.03;1.33] | <0.001 |
| LDL_c (mmol/L) | 2.82 [2.32;3.36] | 2.59 [2.18;3.06] | 2.85 [2.38;3.37] | 2.94 [2.42;3.48] | 2.93 [2.36;3.51] | <0.001 |
| BMI, kg/m^2^ | 23.7 [21.5;26.0] | 20.3 [19.2;21.5] | 22.8 [21.7;23.9] | 24.7 [23.6;25.9] | 27.4 [25.9;29.0] | <0.001 |
| BMI ≥ 25 (n,%) | 91070 (34.8%) | 319 (0.48%) | 5910 (9.03%) | 28348 (43.5%) | 56493 (86.8%) | <0.001 |
| WHtR | 0.49 [0.45;0.53] | 0.43 [0.41;0.44] | 0.47 [0.46;0.48] | 0.51 [0.50;0.52] | 0.56 [0.54;0.58] | <0.001 |

BRI, body roundness index; TyG, triglyceride-glucose; SBP, systolic blood pressure; DBP, diastolic blood pressure; TP, total serum protein; ALB, serum albumin; GLB, serum globulin; BUA, blood uric acid; TBIL, Total Bilirubin; HDL-C, high-density lipoprotein-cholesterol; LDL-C, low-density lipoprotein-cholesterol, WHtR, waist-to-height ratio; BMI, body mass index.

^a^ The survey data was expressed as the median (IQRs) of the continuous variables and the unweighted frequency (weighted proportion) of the categorical variables

**Supplementary Table 2.** Association between the TyG index and periodontitis^1^.

| **Variable** | **Tolerance** | **VIF** |
| --- | --- | --- |
| TyG | 0.58 | 1.72 |
| BRI | 0.42 | 2.39 |
| Sex | 0.48 | 2.39 |
| Age | 0.79 | 1.26 |
| Diabetes | 0.93 | 1.07 |
| HDL-C | 0.61 | 1.65 |
| LDL-C | 0.93 | 1.08 |
| BMI | 0.50 | 1.99 |
| SBP | 0.32 | 3.11 |
| DBP | 0.34 | 2.97 |
| BUA | 0.59 | 1.69 |
| Knowledge | 0.98 | 1.02 |
| Smoking | 0.73 | 1.37 |
| Alcohol consumption | 0.76 | 1.32 |

BRI, body roundness index; TyG, triglyceride-glucose; SBP, systolic blood pressure; DBP, diastolic blood pressure; BUA, blood uric acid; HDL-C, high-density lipoprotein-cholesterol; LDL-C, low-density lipoprotein-cholesterol, BMI, body mass index.

**Supplementary Table 3.** Association between the TyG index and periodontitis^1^.

| TyG | Per 1-SD increment | Groups | | | | p |
| --- | --- | --- | --- | --- | --- | --- |
|  |  | Quartile 1  <8.17 | Quartile 2  8.17-8.59 | Quartile 3  8.59-9.07 | Quartile 4  >9.07 |  |
| Unadjusted | 1.205  (1.187-1.223)*** | 1.000 | 1.230  (1.190-1.270)*** | 1.350  (1.310-1.390)*** | 1.440  (1.400-1.490)*** | <0.001 |
| Model 1 | 1.072  (1.053-1.092)*** | 1.000 | 1.110  (1.070-1.140)*** | 1.130  (1.100-1.170)*** | 1.140  (1.100-1.180)*** | <0.001 |
| Model 2 | 1.075  (1.055-1.096)*** | 1.000 | 1.090  (1.060-1.130)*** | 1.110  (1.070-1.150)*** | 1.130  (1.090-1.170)*** | <0.001 |

^1^Abbreviation: TyG, triglyceride-glucose.

^2^Model 1: adjusted for Sex, Age > 60, diabetes, high-density lipoprotein-cholesterol; Model 2: adjusted for Sex, Age > 60, diabetes, high-density lipoprotein-cholesterol, Knowledge, BMI ≥25, systolic blood pressure, diastolic blood pressure, blood uric acid, low-density lipoprotein-cholesterol, Smoking, Alcohol consumption.

**Supplementary Table 4.** Association between the BRI and periodontitis^1^.

| BRI | Per 1-SD increment | Groups | | | | p |
| --- | --- | --- | --- | --- | --- | --- |
|  |  | Quartile 1  <2.50 | Quartile 2  2.50-3.21 | Quartile 3  3.21-3.96 | Quartile 4  >3.96 |  |
| Unadjusted | 1.176  (1.164-1.187)*** | 1.000 | 1.220  (1.190-1.260)*** | 1.420  (1.370-1.460)*** | 1.620  (1.570-1.670)*** | <0.001 |
| Model 1 | 1.087  (1.075-1.099)*** | 1.000 | 1.120  (1.080-1.160)*** | 1.190  (1.150-1.230)*** | 1.280  (1.240-1.330)*** | <0.001 |
| Model 2 | 1.147  (1.131-1.164)*** | 1.000 | 1.120  (1.090-1.160)*** | 1.260  (1.220-1.310)*** | 1.480  (1.410-1.550)*** | <0.001 |

^1^Abbreviation: BRI, body roundness index.

^2^Model 1: adjusted for Sex, Age > 60, diabetes, high-density lipoprotein-cholesterol; Model 2: adjusted for Sex, Age > 60, diabetes, high-density lipoprotein-cholesterol, Knowledge, BMI ≥25, systolic blood pressure, diastolic blood pressure, blood uric acid, low-density lipoprotein-cholesterol, Smoking, Alcohol consumption.

**Supplementary Table 5.** Association of the combination of the TyG index and BRI with periodontitis risk in individuals^1^.

|  | Groups | | | | p |
| --- | --- | --- | --- | --- | --- |
|  | Low TyG and low BRI  (TyG < 8.60 and BRI < 3.19) | High TyG and low BRI  (TyG > 8.60 and BRI < 3.19) | Low TyG and high BRI  (TyG < 8.60 and BRI > 3.19) | High TyG and high BRI  (TyG > 8.60 and BRI > 3.19) |  |
| Unadjusted | 1.000 | 1.196  (1.162-1.231)*** | 1.380  (1.350-1.411)*** | 1.467  (1.441-1.493)*** | <0.001 |
| Model 1 | 1.000 | 1.066  (1.029-1.105)*** | 1.184  (1.138-1.232)*** | 1.181  (1.135-1.227)*** | <0.001 |
| Model 2 | 1.000 | 1.050  (1.012-1.090)*** | 1.231  (1.171-1.294)*** | 1.243  (1.183-1.305)*** | <0.001 |

^1^Abbreviations: TyG, triglyceride-glucose; BRI, body roundness index.

^2^Model 1: adjusted for Sex, Age > 60, diabetes, high-density lipoprotein-cholesterol; Model 2: adjusted for Sex, Age > 60, diabetes, high-density lipoprotein-cholesterol, Knowledge, BMI ≥ 25, systolic blood pressure, diastolic blood pressure, blood uric acid, low-density lipoprotein-cholesterol, Smoking, Alcohol consumption.

**Supplementary Table 6.** Association of the TyG-BRI with periodontitis risk in individuals^1^.

| TyG-BRI | > 27.83 | Groups | | | | p |
| --- | --- | --- | --- | --- | --- | --- |
|  |  | Quartile 1  <20.725 | Quartile 2  20.725-27.785 | Quartile 3  27.785-35.551 | Quartile 4  >35.551 |  |
| Unadjusted | 1.375  (1.346-1.405)*** | 1.000 | 1.240  (1.200-1.280)*** | 1.450  (1.400-1.490)*** | 1.630  (1.580-1.680)*** | <0.001 |
| Model 1 | 1.151  (1.123-1.179)*** | 1.000 | 1.130  (1.090-1.170)*** | 1.210  (1.170-1.250)*** | 1.280  (1.240-1.330)*** | <0.001 |
| Model 2 | 1.207  (1.172-1.243)*** | 1.000 | 1.130  (1.090-1.170)*** | 1.280  (1.230-1.320)*** | 1.470  (1.400-1.530)*** | <0.001 |

^1^Abbreviations: TyG, triglyceride-glucose; BRI, body roundness index.

^2^Model 1: adjusted for Sex, Age > 60, diabetes, high-density lipoprotein-cholesterol; Model 2: adjusted for Sex, Age > 60, diabetes, high-density lipoprotein-cholesterol, Knowledge, BMI ≥ 25, systolic blood pressure, diastolic blood pressure, blood uric acid, low-density lipoprotein-cholesterol, Smoking, Alcohol consumption.

**Supplementary Table 7.** Association between the TyG, BRI, TyG-BRI and periodontitis^1^.

| Model | Per 1-SD increment | Groups | | | | p |
| --- | --- | --- | --- | --- | --- | --- |
|  |  | Quartile 1 | Quartile 2 | Quartile 3 | Quartile 4 |  |
| TyG | 1.075  (1.054-1.096)*** | 1.000 | 1.091  (1.056-1.128)*** | 1.109  (1.071-1.148)*** | 1.130  (1.088-1.174)*** | <0.001 |
| BRI | 1.142  (1.126-1.159)*** | 1.000 | 1.150  (1.113-1.189)*** | 1.322  (1.272-1.375)*** | 1.503  (1.436-1.572)*** | <0.001 |
| TyG-BRI | 1.014  (1.012-1.015)*** | 1.000 | 1.156  (1.117-1.195)*** | 1.330  (1.279-1.384)*** | 1.492  (1.424-1.564)*** | <0.001 |

^1^Abbreviation: BRI, body roundness index; TyG, triglyceride-glucose.

^2^Model: adjusted for Sex, Age > 60, diabetes, high-density lipoprotein-cholesterol, Knowledge, BMI ≥24, systolic blood pressure, diastolic blood pressure, blood uric acid, low-density lipoprotein-cholesterol, Smoking, Alcohol consumption.

**Supplementary Figure 1.** Univariate logistic regression analysis of periodontitis risk^1^.


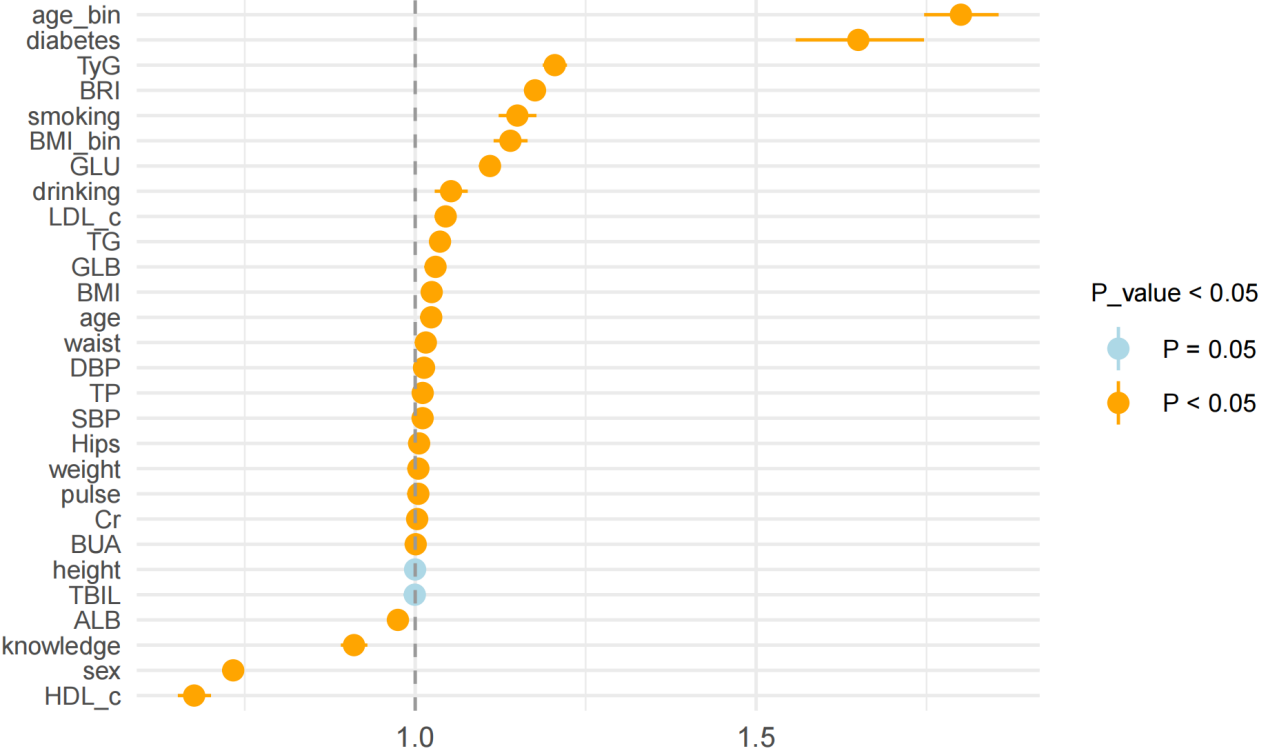
^1^Abbreviations: BRI, body roundness index; TyG, triglyceride-glucose; SBP, systolic blood pressure; DBP, diastolic blood pressure; TP, total serum protein; ALB, serum albumin; GLB, serum globulin; BUA, blood uric acid; TBIL, Total Bilirubin; HDL-C, high-density lipoprotein-cholesterol; LDL-C, low-density lipoprotein-cholesterol; age_bin, age ≥  60; BMI_bin, BMI ≥ 25.

**Supplementary Figure 2.** Univariate logistic regression analysis of periodontitis risk^1^.


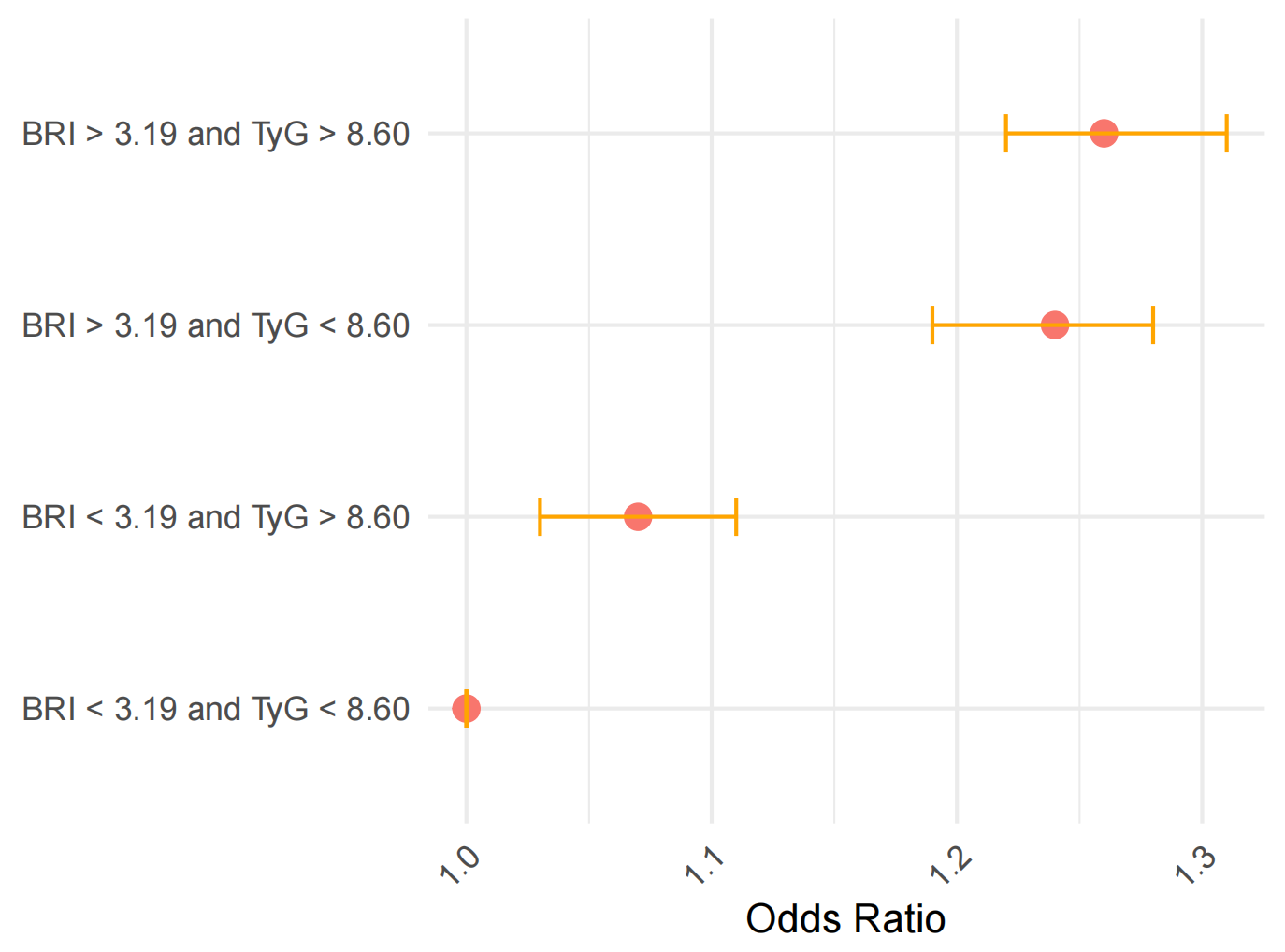


Synergistic effect of the TyG index and BRI on periodontitis risk. BRI, body roundness index; CI, confidence interval; OR, Odds Ratio; TyG, triglyceride-glucose

**Supplementary Figure 3.** Receiver operating characteristic (ROC) curve between triglyceride-glucose (TyG) index and its combined obesity index and the risk of periodontitis.


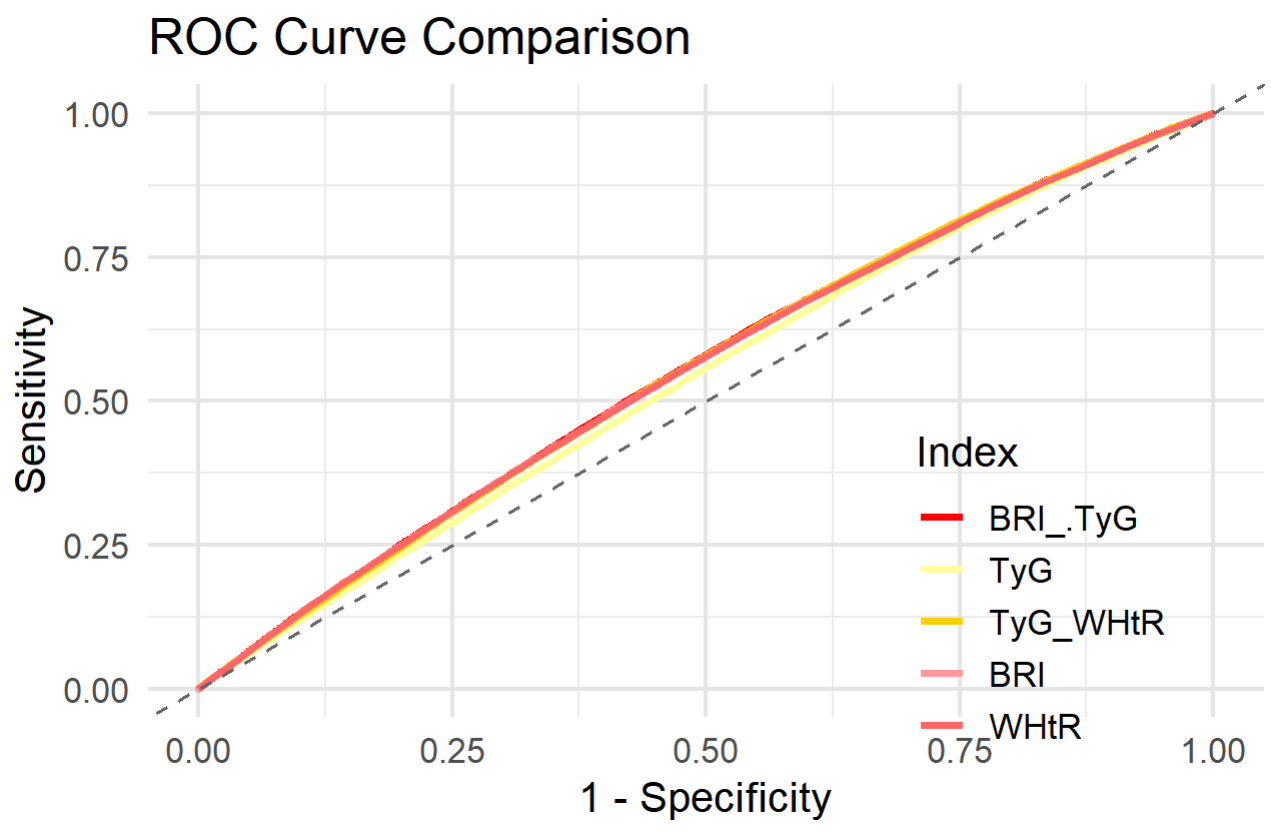

Supplement: Supplementary file 1 [file Table_1.docx]
